# Evaluating a Two-Level vs. Three-Level Fall Risk Screening Algorithm for Predicting Falls Among Older Adults

**DOI:** 10.3389/fpubh.2020.00373

**Published:** 2020-08-13

**Authors:** Thelma J. Mielenz, Sneha Kannoth, Haomiao Jia, Kristin Pullyblank, Julie Sorensen, Paul Estabrooks, Judy A. Stevens, David Strogatz

**Affiliations:** ^1^Department of Epidemiology, Mailman School of Public Health, Columbia University, New York, NY, United States; ^2^Department of Biostatistics, Mailman School of Public Health, Columbia University, New York, NY, United States; ^3^Bassett Research Institute, Center for Rural Community Health, Cooperstown, NY, United States; ^4^The Northeast Center for Occupational Health and Safety in Agriculture, Forestry, and Fishing, Cooperstown, NY, United States; ^5^Department of Health Promotion, College of Public Health, University of Nebraska Medical Center, Omaha, NE, United States; ^6^University of North Carolina Injury Prevention Research Center (UNC IPRC), Carrboro, NC, United States

**Keywords:** falls screening, falls prevention, falls risk, older adults, injury, injury prevention

## Abstract

**Background and Objectives:** Falls account for the highest proportion of preventable injury among older adults. Thus, the United States' Centers for Disease Control and Prevention (CDC) developed the Stopping Elderly Accidents, Deaths, and Injuries (STEADI) algorithm to screen for fall risk. We referred to our STEADI algorithm adaptation as “Quick-STEADI” and compared the predictive abilities of the three-level (low, moderate, and high risk) and two-level (at-risk and not at-risk) Quick-STEADI algorithms. We additionally assessed the qualitative implementation of the Quick-STEADI algorithm in clinical settings.

**Research Design and Methods:** We followed a prospective cohort (*N* = 200) of adults (65+ years) in the Bassett Healthcare Network (Cooperstown, NY) for 6 months in 2019. We conducted a generalized linear mixed model, adjusting for sociodemographic variables, to determine how baseline fall risk predicted subsequent daily falls. We plotted receiver operating characteristic (ROC) curves and measured the area under the curve (AUC) to determine the predictive ability of the Quick-STEADI algorithm. We identified a participant sample (*N* = 8) to gauge the experience of the screening process and a screener sample (*N* = 3) to evaluate the screening implementation.

**Results:** For the three-level Quick-STEADI algorithm, participants at low and moderate risk for falls had a reduced likelihood of daily falls compared to those at high risk (−1.09, *p* = 0.04; −0.99, *p* = 0.04). For the two-level Quick-STEADI algorithm, participants not at risk for falls were not associated with a reduced likelihood of daily falls compared to those at risk (−0.89, *p* = 0.13). The discriminatory ability of the three-level and two-level Quick-STEADI algorithm demonstrated similar predictability of daily falls, based on AUC (0.653; 0.6570). Furthermore, participants and screeners found the Quick-STEADI algorithm to be efficient and viable.

**Discussion and Implications:** The Quick-STEADI is a suitable, alternative fall risk screening algorithm. Qualitative assessments of the Quick-STEADI algorithm demonstrated feasibility in integrating a falls screening program in a clinical setting. Future research should address the validation and the implementation of the Quick-STEADI algorithm in community health settings to determine if falls screening and prevention can be streamlined in these settings. This may increase engagement in fall prevention programs and decrease overall fall risk among older adults.

## Introduction

Falls are the primary cause of injury among older adults, 65 years and older (1–3). Fall-related morbidity, mortality, and institutionalization among older adults cost more than $35 billion annually and are projected to cost $100 billion in 2030 (4, 5). Thus, there is an urgent need to screen for fall risk and intervene to reduce fall incidence through community fall prevention programs (6–8). Falls are not an inevitable part of aging and a number of modifiable risk factors are known (9). The Centers for Disease Control and Prevention (CDC)'s Stopping Elderly Accidents, Deaths and Injuries (STEADI) Tool Kit is intended to help health care providers integrate fall prevention into their practices and target these modifiable risk factors (10, 11). The combination of the STEADI Tool Kit and a plan for fall prevention may reduce fall-related hospitalizations and associated costs (12, 13).

Over the years, the uptake of the STEADI Tool Kit in primary care settings has been slow. In a CDC-funded study, researchers reported on the implementation of the STEADI Tool Kit in a large New York health system, including data from 11 individual healthcare practices. A little over one-third of the clinicians asked their older adult patients if they had fallen in the past year and almost half of these same clinicians reported using performance-based assessments, such as the Timed Up and Go (TUG) test (14). Notably, only one-fifth of providers reported that they referred their older adult participants to a community-based fall prevention program (14). In a commentary, the CDC Injury Center stressed the need for linking clinical care and community-based fall prevention programs, which is described in the STEADI Tool Kit (15, 16).

To increase uptake and scalability of CDC's STEADI Tool Kit in clinical settings, we adapted the CDC's STEADI algorithm for fall risk screening by having trained paraprofessionals conduct a modified screening among older adults. We refer to this adapted STEADI screening as the “Quick-STEADI” algorithm. The “Quick-STEADI” algorithm determines older adults' fall risk based on their responses to three key questions regarding past year falls, concerns about falling, and balance problems. In addition, the algorithm considers participants' individual TUG test scores, which provide an objective assessment of one's gait, strength, and balance.

The CDC's STEADI algorithm originally screened participants into three fall risk categories (low, moderate, and high), but transitioned more recently to using only two fall risk categories (not at-risk and at-risk). The purpose of this study was to implement and assess our newly developed Quick-STEADI algorithm for fall risk screening in a sample of older adults in a rural clinic using the RE-AIM framework. We evaluated the association between the two- vs. three-level fall risk categories and subsequent falls. We also compared the discriminatory ability of Quick-STEADI for these three- vs. two-level fall risk categories. Furthermore, we evaluated the qualitative implementation of the Quick-STEADI algorithm in clinical settings.

The Quick-STEADI algorithm may help promote widespread adoption of the CDC's STEADI Tool Kit by using a train-the-trainer model to conduct fall risk assessment by paraprofessionals, provide information about evidence-based community fall prevention programs, and promote communication with older adults' health care providers.

## Materials and Methods

### Study Design and Sample

The RE-AIM planning and evaluation framework was used to define outcomes related to participant-level benefits (e.g., reach and effectiveness of Quick-STEADI) and organizational considerations for uptake [e.g., potential for adoption, implementation, and sustainability; (17, 18)]. Quantitative data were used to document study reach (number, proportion, and representativeness of the study sample to the intended audience) and fall risk screening effectiveness (ability of Quick-STEADI to distinguish between levels of fall risk). Qualitative interviews assessed participant perceptions of the Quick-STEADI screening process. Assessing participants' sustained benefits was beyond the scope of this evaluation (e.g., individual-level maintenance). Qualitative data were collected using a focus group of staff members that implemented Quick-STEADI to evaluate adoption, implementation, and maintenance.

The prospective cohort study sample consisted of 200 individuals, 65 years and older, recruited from the Bassett Healthcare Network (BHN) in Cooperstown, New York. At baseline, the majority of Quick-STEADI fall risk screenings took place in waiting area of the BHN primary care clinics. Patients who had come in for appointments were approached to determine their interest and eligibility for the study. An informed consent process was completed with signed consent forms before any data were collected. The completion of baseline three-level fall risk screening was intended to be recorded in real time on a tablet (e.g., iPads) to immediately generate a risk score for the participant. After completing baseline fall risk screening, study participants were then followed for 6 months to assess incidence of daily falls. We chose to follow participants for 6 months, given that existing literature in falls epidemiology have previously used 6 months as a follow-up period to determine the validation of fall risk screening algorithms (19, 20) and a 6-month follow-up period additionally provided increased feasibility.

During the 6-month follow-up period, study staff delivered an online weekly falls calendar to participants via email on each Monday, so that participants could track any incident falls for the previous week. For each day, the participant would click a button to indicate whether they “fell” or “did not fall” on that day. If a person experienced a fall, he or she was prompted to describe the circumstances of the fall. Study staff also provided paper falls calendars, if the participant did not have access to internet, a computer/tablet, or other technological resources. These paper fall calendars were mailed out a few weeks after baseline screening was complete and again at the end of the study if their falls calendar data were not completed. These paper calendars were set up in an identical manner to the online calendars.

At the end of the 6-month follow-up period, participants reported whether they had chosen to partake in interventions that focus on fall prevention. All participants had the opportunity to partake in fall prevention programs and thus, interventions were not dependent on the outcome of the baseline fall risk screening algorithm. Given that few participants (*N* = 3) had partaken in fall prevention programs, we did not consider possible effects that preventive interventions may have had on subsequent daily fall incidence of participants.

The study protocol was reviewed and approved by the Columbia University Institutional Review Board [AAAR6554] and the Mary Imogene Bassett Hospital Institutional Review Board [2094].

### Measures

#### Exposures

At baseline, participants completed a demographics questionnaire, Stay Independent Checklist, Falls Efficacy Scale, and the Quick-STEADI algorithm for fall risk screening. Existing literature demonstrate that the Stay Independent Checklist and the Falls Efficacy Scale yield strong predictive validity and thus, for the purpose of this study, we will be assessing the predictive validity of the Quick-STEADI algorithm (21, 22).

The three-level Quick-STEADI algorithm consisted of three key questions (regarding falls in the past year, concerns about falls, and balance issues), TUG test results (including observations of gait and balance), fall count in the past year, and injury count in the past year. The results were used to determine if the patient was at low, moderate, or high risk for falls.

The two-level Quick-STEADI algorithm consisted of three key questions (regarding falls in the past year, concerns about falls, and balance issues) and the TUG test results (including observations of gait and balance). The results were used to determine if the patient was at risk or not at risk for falls.

**Figure 1:**
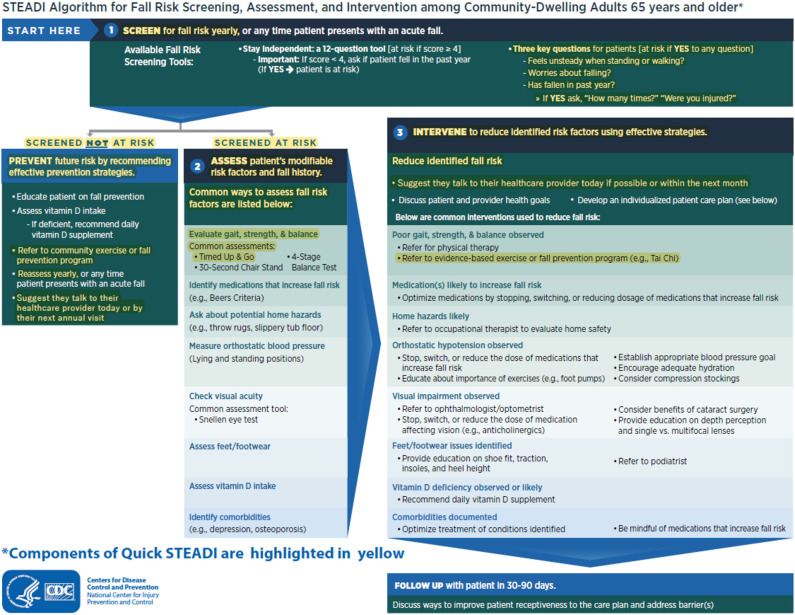
Two-level CDC STEADI algorithm flowchart.

**Figure 2:**
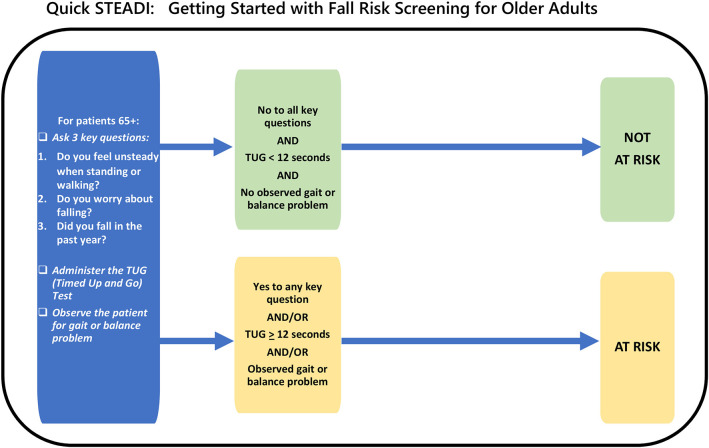
Adapted two-level Quick-STEADI algorithm flowchart.

To demonstrate how the two-level Quick-STEADI algorithm was adapted from the CDC STEADI algorithm, we have provided a visual in which the components of the two-level Quick-STEADI algorithm are highlighted in yellow in the current CDC STEADI algorithm for fall risk screening (Figure 1). A directional flowchart illustrates the steps of the adapted two-level Quick-STEADI screening procedure used in this study (Figure 2).

#### Outcomes

During the 6-month follow-up period in our study, participants reported whether or not they fell on each day using a weekly falls calendar. Daily fall outcomes (dichotomized as yes or no) were the primary outcome of the study.

If participants had fallen on a particular day, they also provided relevant characteristics of the specific fall event, in that participants reported the type of injury that they had experienced (no injury at all; minor injury; moderate injury; serious injury), the location of the fall (floor or level ground; stairs, steps or escalator; curb, including sidewalk; chair, bed, sofa, or other furniture; other location). If participants reported other type of fall, then they would provide an open-ended response to specify the location that they had fallen in. Participants also reported the time of fall (morning; afternoon; evening; night) and the cause of fall (slipped or tripped; misplaced step; loss of balance; legs gave way; other cause). If participants reported other type of cause for the fall, then they would provide an open-ended response to specify the cause of their fall. At the end of 6 months, participants reported whether they had attended a fall prevention program before, during, and after the study period. Participants then had an opportunity to provide an open-ended response to specify the name of the fall prevention program, the number of sessions that they had attended, and the location of the fall prevention program.

#### Covariates

We adjusted for sociodemographic variables measured at baseline, including: age (continuous, measured in years), sex (male; female), and education (less than high school; high school or vocational school; some college or higher). We collected data regarding these potential sociodemographic confounders, given that existing falls screening validation research adjusted for similar covariates (11).

### Statistical Analyses

We first examined the distribution of the sociodemographic and health characteristics in the total study sample. We then assessed the distribution of sociodemographic and health characteristics among those who were classified as low risk, moderate risk, and high risk, using the three-level Quick-STEADI fall risk screening algorithm, and among those who were classified as at-risk or not-at risk, using the two-level Quick-STEADI fall risk screening algorithm (Table 1).

**Table 1:** Characteristics of Quick-STEADI Participants.

	**Total sample**	**By Quick-STEADI three risk level**			**By Quick-STEADI two risk level**	
	***N* = 200**	**Low risk *N* = 61**	**Moderate risk *N* = 88**	**High risk *N* = 48**	**Not at risk *N* = 47**	**At risk *N* = 150**
**Gender**						
Female	112 (56.00%)	32 (52.46%)	52 (59.09%)	26 (54.17%)	22 (46.81%)	88 (58.67%)
**Age**	74.78 (6.50)	73.03 (5.98)	75.74 (6.40)	74.48 (6.46)	72.60 (5.62)	75.22 (6.47)
**Race**						
White/Caucasian	197 (98.50%)	60 (98.36%)	88 (100.00%)	46 (95.83%)	47 (100.00%)	147 (98.00%)
American Indian	1 (0.50%)	0 (0.00%)	0 (0.00%)	1 (2.08%)	0 (0.00%)	1 (0.67%)
Alaskan Native	1 (0.50%)	1 (1.64%)	0 (0.00%)	0 (0.00%)	0 (0.00%)	1 (0.67%)
Other	1 (0.50%)	0 (0.00%)	0 (0.00%)	1 (2.08%)	0 (00.00%)	1 (0.67%)
**Ethnicity**						
Non-Hispanic	196 (98.00%)	61 (100.00%)	87 (98.86%)	45 (93.75%)	47 (100.00%)	146 (97.33%)
Hispanic	4 (2.00%)	0 (0.00%)	1 (1.14%)	3 (6.25%)	0 (0.00%)	4 (2.67%)
**Education**						
Less than high school	6 (3.00%)	1 (1.64%)	5 (5.68%)	0 (0.00%)	1 (2.13%)	5 (3.33%)
High school and vocational school	61 (30.50%)	19 (31.15%)	25 (28.41%)	17 (35.42%)	14 (29.79%)	47 (31.33%)
Some college or higher	135 (66.50%)	41 (67.21%)	58 (65.91%)	31 (64.58%)	32 (68.09%)	98 (65.33%)
**Marital status**						
Partner	143 (72.22%)	46 (76.67%)	62 (71.26%)	33 (68.75%)	33 (71.74%)	108 (72.48%)
No partner	55 (27.78%)	14 (23.33%)	25 (28.74%)	15 (31.25%)	13 (28.26%)	41 (27.52%)
**Physical health**						
**Current eyesight (with corrective lenses)**						
Excellent	26 (13.20%)	4 (6.56%)	14 (16.28%)	8 (17.02%)	4 (8.51%)	22 (14.97%)
Very good	66 (33.50%)	25 (40.98%)	29 (33.72%)	11 (23.40%)	22 (46.81%)	43 (29.25%)
Good	68 (34.52%)	24 (39.34%)	27 (31.40%)	15 (31.91%)	16 (34.04%)	50 (34.01%)
Fair	29 (14.72%)	7 (11.48%)	14 (16.28%)	8 (17.02%)	4 (8.51%)	25 (17.01%)
Poor	6 (3.05%)	0 (0.00%)	1 (1.16%)	5 (10.64%)	0 (0.00%)	6 (4.08%)
Legally blind (volunteered)	2 (1.02%)	1 (1.64%)	1 (1.16%)	0 (0.00%)	1 (2.13%)	1 (0.68%)
**Assistive walking device use (e.g., cane, walker, etc.)**						
Yes	38 (19.00%)	3 (4.92%)	18 (20.45%)	17 (35.42%)	1 (2.13%)	37 (24.67%)
**Hip, knee, ankle, foot surgery in past year**						
Yes	10 (5.00%)	0 (0.00%)	7 (7.95%)	3 (6.25%)	0 (0.00%)	10 (6.67%)
**Medication use for sleep or mood enhancement**						
Yes	49 (24.75%)	12 (19.67%)	17 (19.54%)	19 (40.43%)	6 (12.77%)	30 (20.55%)

We additionally evaluated the participants' fall count (0, 1, 2+) and whether participants experienced an injurious fall (no; yes) among those who were classified as low risk, moderate risk, and high risk, using the three-level Quick-STEADI fall risk screening algorithm and among those who were classified as at-risk or not at-risk, using the two-level Quick-STEADI fall risk screening algorithm (Table 2).

**Table 2:** Number of falls and injurious falls during the follow-up time.

	**Three-level Quick-STEADI**			**Two-level Quick-STEADI**		**Total**
	**Low**	**Moderate**	**High**	**Not at risk**	**At risk**	
**Falls count**						
0	45 (84.91%)	61 (85.92%)	32 (74.42%)	35 (89.74%)	103 (80.47%)	138
1	7 (13.21%)	8 (11.27%)	6 (13.95%)	3 (7.69%)	18 (14.06%)	21
2+	1 (1.89%)	2 (2.82%)	5 (11.63%)	1 (2.56%)	7 (5.47%)	8
**INJ. Falls**						
No	49 (92.45%)	63 (88.73%)	35 (81.40%)	36 (92.31%)	111 (86.72%)	147
Yes	4 (7.55%)	8 (11.27%)	8 (18.60%)	3 (7.69%)	17 (13.28%)	20
Total	53	71	43	39	128	167

To evaluate the discriminatory ability of the two- vs. three-level risk categories in the Quick-STEADI algorithm, we used receiver operating characteristic (ROC) curves to compare the area under the curves (AUC).

The following equations were used for the statistical analysis:

*p*: probability of a fall in each day, modeled using a logistic regression model: $log\left(\frac{p}{1-p}\right)=\beta x$, where *x* is a vector of predictors, including two or three level risk scores from the Quick-STEADI algorithm.

*n*_*i*_: number of follow-up days for a specific participant.

θ_*i*_: probability of a specific participant having at least one fall during their respective follow-up period. Given that participants yielded different follow-up periods, the probability of participants having at least one fall during follow-up is:

$$\begin{array}{l} {\hat{\theta }}_{i}=1-{\left(\frac{1}{1+\exp\left(\beta x_{i}\right)}\right)}^{n_{i}} \end{array}$$

AUC of ROC was calculated based on observed falls and predicted risk of falls, ${\hat{\theta }}_{i}$, at various threshold settings for each participant. Generalized linear mixed models (GLMM) were also used to examine the daily fall risk for the Quick-STEADI algorithm two- vs. three-level fall risk categories, after adjusting for sociodemographic characteristics. We used SAS 9.4 statistical software to perform all analyses.

### Qualitative Assessment

To conduct a qualitative assessment of the program, a convenience sample of eight participants (3 male and 5 female) was identified from a sampling frame of 25 individuals who had completed the baseline data collection. All participants in the convenience sample had completed the Quick-STEADI fall risk screening and follow-up. Study team members contacted these individuals by phone to assess their interest in taking part in an interview to discuss their participation in Quick-STEADI. Two evaluation team members (KP and JS) then conducted participant interviews either over the phone or in person, based on the individual's preference. Questions in the interviewer guide were developed collectively by the study team and focused on the participant's experiences with the screening process, engagement with the falls calendar, and follow-up with local fall prevention programming. Novel themes or commentary ceased to emerge by the eighth interview, ensuring that saturation had been achieved.

In addition to the participant interviews, the qualitative assessment of the program included a focus group conducted with three screeners who were responsible for recruiting participants at the BHN primary care clinics. The focus group was designed to elicit feedback on the implementation of the screening process and the degree of engagement by healthcare professionals at BHN to better understand the potential for future adoption, implementation, and sustainability.

**Figure 3:**
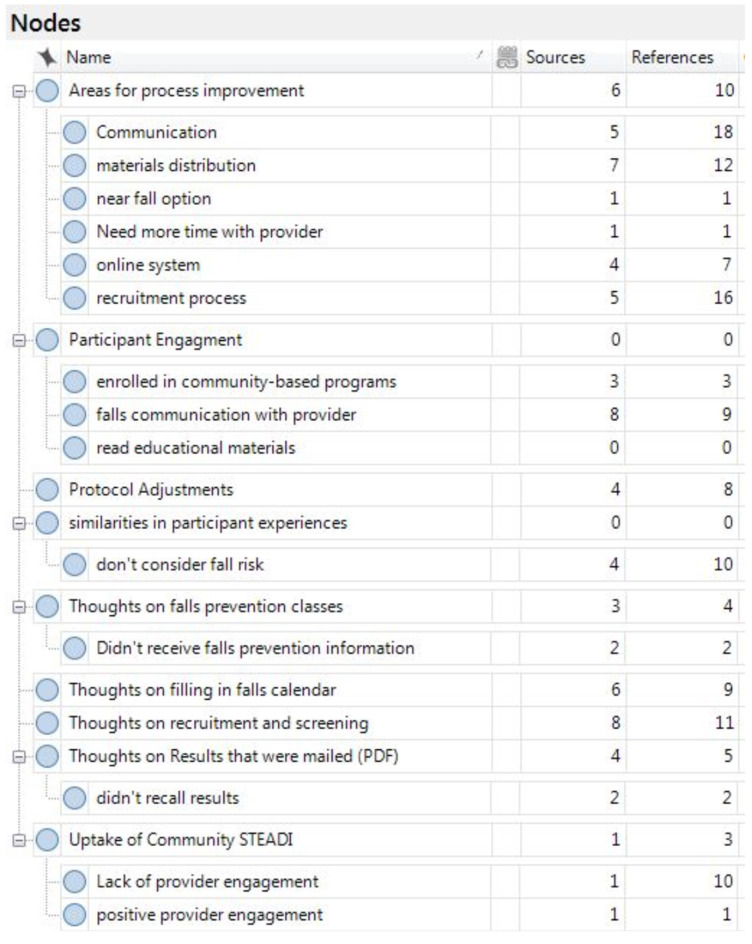
Subcategories of *a priori* codes for thematic analysis of the focus groups.

All interviews and focus groups were tape-recorded and transcribed. Transcriptions were uploaded into NVIVO for thematic analysis. Prior to coding, KP read the transcriptions and identified any potential transcription errors. Initial *a priori* codes were developed by JS and KP to evaluate the implementation of the Quick-STEADI screening at BHN. As coding proceeded, *a priori* codes were grouped to create categories and subcategories. These were revised continuously to better reflect the experiences of the participants and to better inform the primary research questions driving the qualitative evaluation. Final categories and subcategories are provided in Figure 3.

## Results

### Quantitative Data Analysis Results

#### Reach

Approximately 500 BHN patients were exposed to recruitment activities resulting in a study sample of 200 participants and yielding a 40% participation rate. Of these 200 older adults, 56% were female, 98.5% were white, and 98.0% were non-Hispanic, 3.0% had not completed high school, 30.5% were high school graduates, and 66.5% had at least some college education. When compared to the characteristics of the BHN patient population (50.4% female; 90.5% white; 95% non-Hispanic; 11.6% completed less than high school, 35% high school graduates, and 53.4% completed at least some college), the study sample participants were fairly representative of the BHN patient population, with only minor differences as slightly more individuals were female, white, non-Hispanic and more educated in the study sample. Table 1 presents the sociodemographic and physical health characteristics of the study sample in further detail.

#### Effectiveness

As shown in Table 2, the number of falls did not differ significantly between the low, moderate, and high-risk levels for the three-level Quick-STEADI algorithm (*p* = 0.17) and between the at-risk and not at-risk levels for the two-level Quick-STEADI algorithm (*p* = 0.41). The number of injurious falls also did not differ significantly between the low, moderate, and high-risk levels for the three-level Quick-STEADI algorithm (*p* = 0.25) and between the at-risk and not at-risk levels for the two-level Quick-STEADI algorithm (*p* = 0.35).

**Figure 4:**
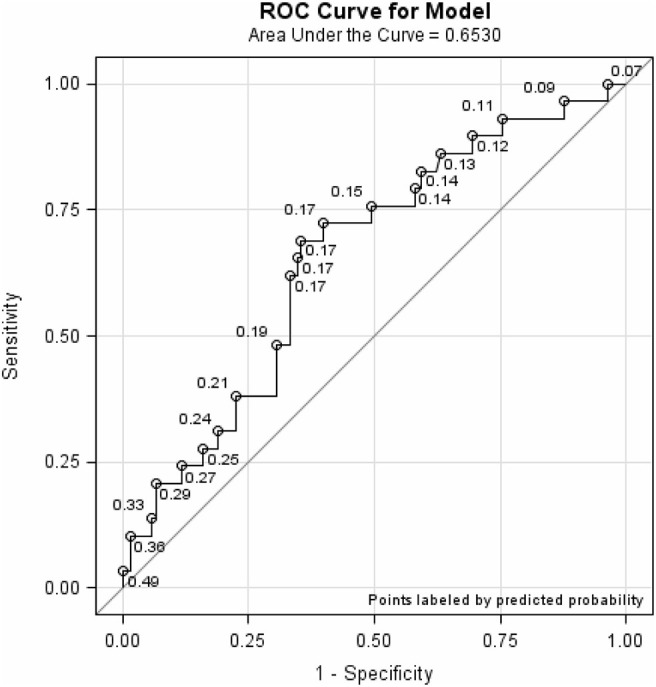
ROC curve demonstrating predictive validity of the three-level Quick-STEADI algorithm for fall risk, adjusted for age, sex, and education.

**Figure 5:**
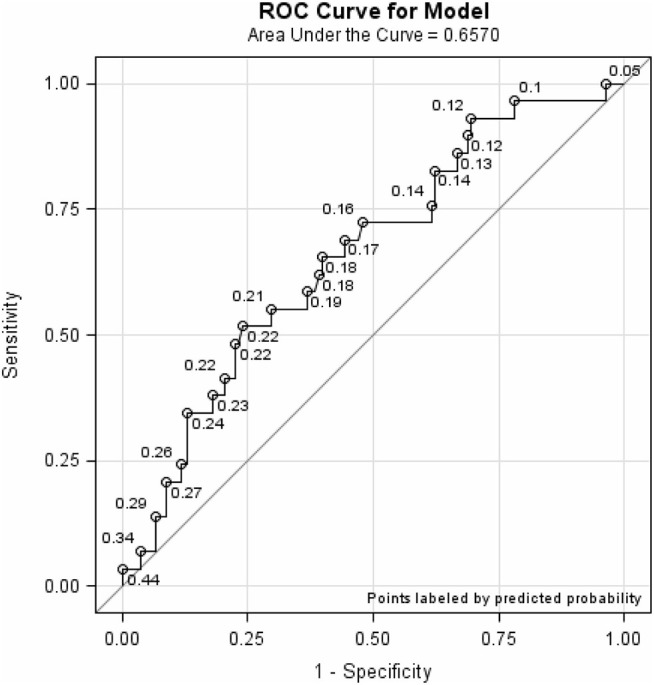
ROC curve demonstrating predictive validity of the two-level Quick-STEADI algorithm for fall risk, adjusted for age, sex, and education.

The AUC for the discriminatory ability of the three-level and two-level Quick-STEADI algorithms and selected demographics (age, sex, and education) yielded values of 0.653 and 0.657, respectively. Figures 4, 5 show the ROC curves for the three- and two-level Quick-STEADI algorithms.

Table 3 demonstrates the GLMM findings for the three-level and the two-level Quick-STEADI algorithms. Participants in the low and moderate risk levels were, respectively, associated with a reduced risk of daily falls compared to those in the high-risk level (−1.09, *p* = 0.04; −0.99, *p* = 0.04). Participants in the not at-risk level were not associated with a reduced risk of daily falls compared to those in the at-risk level (−0.89, *p* = 0.13).

**Table 3:** Generalized linear model estimates for prediction of daily fall risk by the two- and three-level Quick-STEADI algorithm, adjusting for age, sex, and education.

**Effect**	**Estimate**	**Standard error**	**DF**	***t-*Value**	***Pr > | t |***
**Three-level**					
Low	−1.0912	0.5263	160	−2.07	0.0397*
Moderate	−0.9988	0.4878	160	−2.05	0.0422*
High	0				
**Two-level**					
Not at risk	−0.8863	0.5791	161	−1.53	0.1279
At risk	0				

* *p < 0.05*.

### Qualitative Data Analysis Results

#### Feedback on the Quick-STEADI Recruitment and Screening Process

In the interviews, participants stated that they felt comfortable being screened in these settings and that these were good places to reach people to conduct Quick-STEADI. Several participants said it would have been helpful to have a CDC STEADI Tool Kit poster displayed in the waiting area so they would know what to expect and to make it clear that the health system was supporting this effort. The timing of the Quick-STEADI implementation was raised as an issue. Specifically, the focus group reported that the screening should not take place while participants were waiting for their appointments, as some participants did not complete the process because the screening was interrupted by their appointments. In addition, screeners felt rushed because they did not know how much time they had, which may have interfered with implementation. Overall, both participants and screeners felt that the screening process itself was relatively quick, non-invasive, and could be adopted for use in other similar settings.

#### Feedback on the Quick-STEADI Data Collection Tools and Process

The focus group with Quick-STEADI screeners found that the online system was cumbersome and difficult to navigate, especially when there was time pressure to complete the screening. Screeners felt that paper and pen were easier to use.

The members of the screener focus group also identified important aspects regarding adoption and implementation, related to the multiple stakeholders within the health system. First, there was some question about the degree to which providers were aware of the CDC STEADI Tool Kit or were engaged in the implementation process. The results of the focus group indicated that providers needed increased awareness of the Quick-STEADI screening implementation to maximize effectiveness. Second, the focus group reported that the Quick-STEADI screening was relatively easy to implement and that it could be successfully employed in their network. Third, multi-leveled support and action was necessary to increase the likelihood of sustained implementation. For example, the screener focus group suggested that it was necessary to have buy-in from the front-line staff, providers, and the medical administration. Without buy-in at each level, the likelihood of implementation and sustainability would be reduced.

#### Participant Feedback on Falls Calendar Recording

Based on feedback provided in the participant interviews, online users were somewhat confused about the source of the email because it did not contain a BHN email address. One participant suggested that a preliminary email notice may have prevented them from deleting the first two or three reminders. For the most part, the online users thought that filling in the falls calendar was “quick” and “easy.” However, the size of the screen was small, especially if participants were using their mobile interfaces and there was some difficulty enlarging the screen. The screeners focus group offered little detail regarding the ease of the online falls calendar. This is understandable since they were not involved in this aspect of the implementation.

For these participants, completing the calendars proved to be arduous. One popular sentiment was that instead of filling in the “fall” or “did not fall” bubbles each day, it would be easier to note only the days in which they fell. One participant stated, “I would just have one sheet on there that would explain that if you have fallen, put the date, time and why.” Another pointed out that there was no “near fall” option on the calendar, which would have been helpful.

## Discussion

This study sought to modify CDC's STEADI algorithm for fall risk screening to allow for broader implementation by paraprofessionals. Our goal was to increase the uptake of CDC's STEADI Tool Kit by primary care clinics and increase older adults' involvement in evidence-based community fall prevention programs. Regarding the validation assessment of the Quick-STEADI screening implementation, our findings demonstrated that the two-level Quick-STEADI fall risk screening algorithm (AUC: 0.653) and the three-level Quick-STEADI fall risk screening algorithm (AUC: 0.657) yielded moderate predictive validity. Both algorithms identified many false positives, in classifying more participants who did not experience a fall event, as at risk for falls. Furthermore, our results exhibited that using two-level fall risk categories was as accurate at predicting future fall events as using the three-level fall risk categories, given that the two-level Quick-STEADI algorithm yielded similar AUC values as the three-level Quick-STEADI algorithm. It is additionally important to note that the adjusted GLMM analysis indicated that only the three-level Quick-STEADI algorithm yielded statistical significance in predicting daily falls and the two-level Quick-STEADI algorithm did not yield statistical significance in predicting daily falls.

We found that our study findings supported existing literature in falls epidemiology. The two-level Quick-STEADI fall risk screening algorithm (AUC: 0.65) yielded similar predictive validity as the three-level STEADI algorithm (AUC: 0.64), assessed in contemporary falls validation research (11). Thus, current fall risk screening algorithms identify a high proportion of false positives; thereby, contributing to a potential misuse of intervention resources. Nonetheless, it is likely that increased false positives may not be a limitation, given that falls interventions, such as physical activity promotion, are relatively low-cost (23).

Results from the qualitative assessment of the Quick-STEADI screening at BHN illustrate that it is possible to implement a fall risk screening program in a rural healthcare network setting. These findings are aligned with existing literature that have also determined fall risk screening algorithms to be feasible and easy to use within clinical and community-wide settings (24, 25). Furthermore, participant and screener focus groups provided specific suggestions to refine and improve Quick-STEADI's implementation and patient engagement: (1) Screenings should not take place while participants are waiting to see their providers. (2) Falls calendar emails should clearly come from the health provider, display a large screen and focus on highlighting only days with a fall (instead of tracking all days) to decrease individual burden. (3) Front line providers and staff must be engaged in recruiting Quick-STEADI participants. Clinics should prominently exhibit information about the program as well as a clear schedule of the times that Quick-STEADI screeners will be present. In this way, providers would know when to alert their eligible participants about the opportunity for screening after their appointment.

Study limitations included a lack of racial diversity within the study sample. Participants were predominantly white, although the cohort was balanced across gender and age. Furthermore, the population was rural and had a higher fall rate than the older population state-wide, indicating a potential lack of generalizability, but a need for increased falls screening in this particular population. The study findings may also not be generalizable to diverse sub-groups outside of the Bassett Healthcare Network in Cooperstown, New York. A larger sample size would have provided increased study power. However, the relatively moderate AUC values could not be attributed to a lack of power, given that a fall is a random event. Thus, it is difficult to predict if a participant would have a fall at a given time point and increasing the sample size would not resolve this issue. Lastly, participants who chose to partake in fall prevention programs during the study, may have experienced less subsequent daily falls. Nonetheless, we believe that this would not be a major concern, given that few participants (*N* = 3) reported that they had engaged in a fall prevention program.

Additional research is needed regarding the CDC's STEADI algorithm, to evaluate if fall risk screening should return to the three levels or maintain the current two levels. Although Quick-STEADI was implemented in a wellness setting, it may be feasibly used in community health settings to facilitate the screening process. Further research should continue to evaluate the feasibility of implementing the Quick-STEADI algorithm in community health settings to determine if the modified algorithm is an optimal fall risk screening tool. Promoting the accessibility of the CDC's STEADI Tool Kit in community wellness settings may also increase utilization rates of fall prevention programs. Providing greater access to falls intervention resources can help reduce fall risk among older adults and curb the steep healthcare costs associated with fall-related injuries.

Future studies will be able to utilize Quick-STEADI in the *Epic* foundation system that was built alongside this pilot. Many of the limitations identified in the qualitative feedback will be rectified with Quick-STEADI residing in *Epic*. The system will not be difficult to navigate for the screeners, ongoing falls information will be emailed to them through MyChart, which will be recognizable to the patient and be more streamlined than the falls calendars used in this study. The CDC's STEADI already exists in *Epic's* foundation system titled “Preventing Falls in Primary Care Using STEADI” (26). Furthermore, the Quick-STEADI screening process seemed to appeal to the participants and showed promise in its ability to reach and benefit the intended audience.

In summary, our RE-AIM evaluation provides preliminary evidence that the Quick-STEADI algorithm for fall risk screening can reach older adults and effectively identify those who would benefit from fall prevention activities and programs (27). Participants provided qualitative feedback on its accessibility and ease of use. Screeners who implemented Quick-STEADI felt that it could be adopted, implemented, and sustained in community health settings—with the caveat that a systems-based implementation approach was necessary to ensure buy-in from staff, providers, and organizational leaders (28).

## Data Availability Statement

The raw data used to support the findings of this study are restricted by the Columbia University Institutional Review Board in order to protect research subject privacy.

## Ethics Statement

The studies involving human participants were reviewed and approved by both the Columbia University Institutional Review Board [AAAR6554] and the Mary Imogene Bassett Hospital Institutional Review Board [2094]. The patients/participants provided their written informed consent to participate in this study.

## Author Contributions

TM: conceptualization and supervision. TM, SK, HJ, KP, JSo, PE, JSt, and DS: methodology, writing—review, and editing. TM, HJ, SK, KP, and JSo: formal analysis. TM, SK, HJ, KP, JSo, and DS: data curation. TM and SK: writing—original draft preparation. All authors have read and agreed to the published version of the manuscript.

## Conflict of Interest

The authors declare that the research was conducted in the absence of any commercial or financial relationships that could be construed as a potential conflict of interest.
